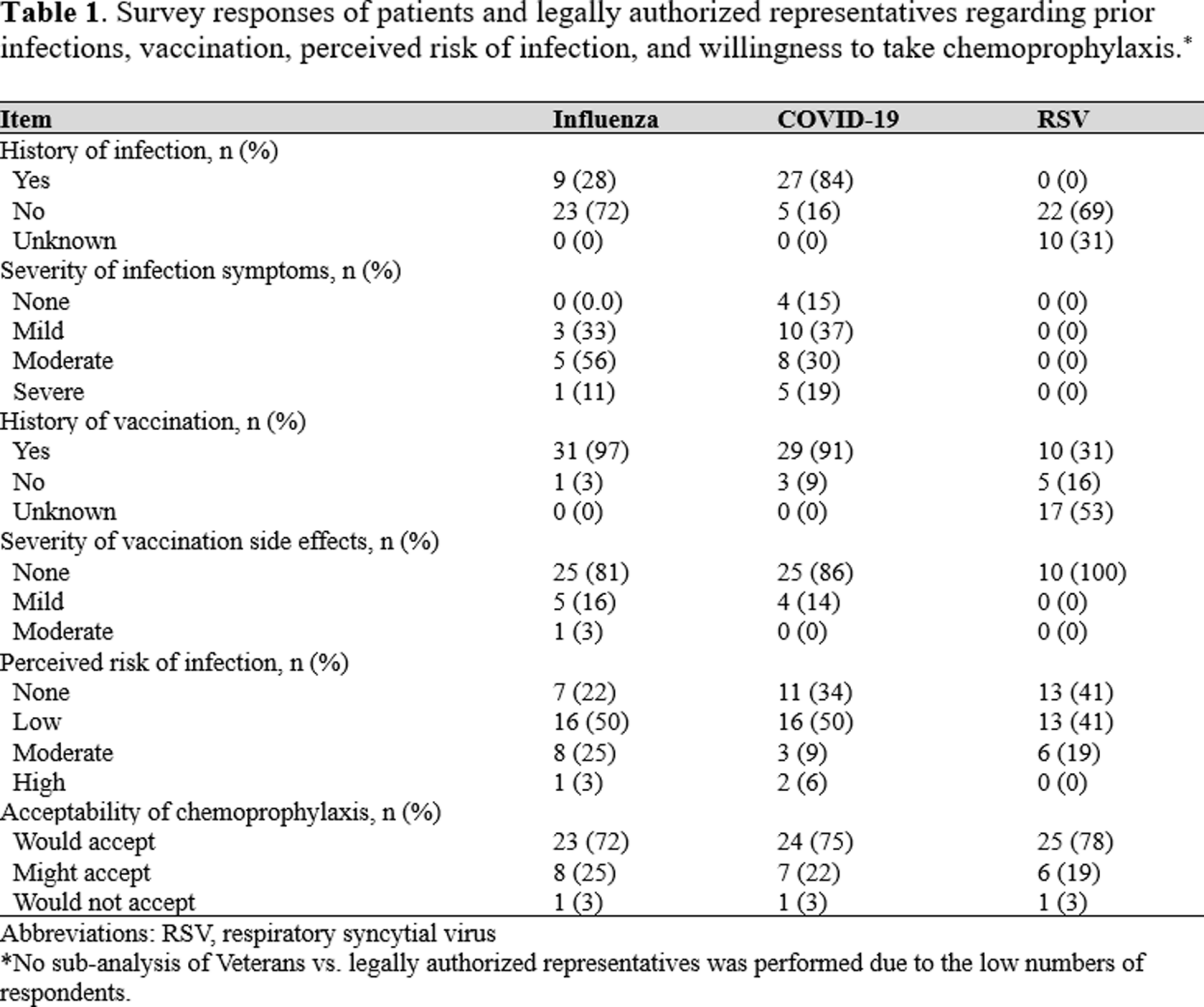# 281 Urine Collection Practices in Catheterized Adults: Implications for UTI Classification and Antibiotic Use

**DOI:** 10.1017/ash.2026.10642

**Published:** 2026-06-23

**Authors:** Alison O’Donnell, Shaye Kerper, Mary Connolly, Robin Jump

**Affiliations:** 1 Veteran’s Administration Pittsburgh Health System (VAPHS); 2 University of Pittsburgh; 3 UPMC; 4 VA Pittsburgh Healthcare System

## Abstract

**Background:** Post-acute and long-term care (PALTC) residents are disproportionately affected by viral infections such as influenza, COVID-19, and respiratory syncytial virus (RSV). While chemoprophylaxis is the standard of care for mitigating the spread of influenza in PALTC settings, there are no agents available for chemoprophylaxis against outbreaks of COVID-19 or RSV at present. However, as we advance in our knowledge of antivirals, agents for chemoprophylaxis against outbreaks of COVID-19 and RSV may be available in the future. Little is known about the perspectives of PALTC residents and their care partners regarding the use of chemoprophylaxis against respiratory viruses. **Methods:** From November 2024 to July 2025, we surveyed residents at a 152-bed community living center (CLC). Inclusion criteria were CLC residents age > **Results:** Out of 81 eligible residents and LARs, 40% (N=32; 6 residents and 26 LARs) completed the survey. Respondents indicated 9 (28%) and 27 (84%) of residents had an influenza or COVID-19 infection, respectively, during their PALTC stay (Table 1). None recalled an RSV infection though 29 (91%) respondents had previously heard of RSV. Most respondents reported that residents had been vaccinated against influenza (n=31, 97%) and COVID-19 (n=29, 91%), while only 10 (31%) reported that residents had been vaccinated against RSV. Most residents or LARs, on behalf of their resident, indicated they would accept chemoprophylaxis during an outbreak of influenza (n=23, 72%), COVID-19 (n=24, 75%), or RSV (n=25, 78%). Only one resident indicated he would decline chemoprophylaxis for all three viruses. **Conclusions:** The majority of Veterans, or their representatives, in the PALTC setting would consent to chemoprophylaxis for influenza, COVID-19, and RSV if it were available and recommended by a healthcare provider. Gaining a better understanding of how PALTC residents and their care partners perceive the use of chemoprophylaxis against respiratory viruses can support the effective implementation of these programs.

**Figure:**